# Physician referrals of patients with neck and low back pain for physical therapy in outpatient clinics: a cross-sectional study

**DOI:** 10.1186/s13584-025-00683-7

**Published:** 2025-04-03

**Authors:** Amira Daher, Gali Dar

**Affiliations:** 1 Department of Physical Therapy, Faculty of Social Welfare & Health Sciences, University of Haifa, Haifa, Israel; 2 Department of Physical Therapy, Faculty of Health Studies, Zefat Academic College, Zefat, Israel; 3 Department of Health Systems Administration, Max Stern Academic College of Emek Yezreel, Yezreel Valley, Israel; 4 Physical Therapy Clinic, The Ribstein Center for Sport Medicine Sciences and Research, Wingate Institute, Netanya, Israel

**Keywords:** Healthcare services planning, Musculoskeletal disorders, Neck pain, Low back pain, Physical therapy referrals, Age differences, Sex differences

## Abstract

**Background:**

Patients commonly seek outpatient physical therapy services for musculoskeletal disorders. Understanding these patient groups in Israel provides valuable insights into the healthcare system. We aimed to investigate physician referral patterns for physical therapy across different age and sex groups, focusing on neck and low back pain. Additionally, we sought to explore the therapeutic interventions provided by physical therapists for these conditions.

**Methods:**

For this retrospective, cross-sectional study we utilized data from a national health maintenance organization covering > 4 million people at 100 physical therapy outpatient clinics. We measured the prevalence rates of physicians’ referral patterns for neck and low back pain according to age and sex, as well as therapeutic interventions prescribed by physical therapists. We used Z-tests to assess the differences in prevalence rates between women and men within the same age group. Logistic regression analyses were used to evaluate the likelihood of patients of a specific age group being referred to physical therapy compared with the total sample. We analyzed prevalence rates of different treatment protocols used by physical therapists according to these referrals.

**Results:**

Overall, 1,593,592 physician referrals for physical therapy were made over 6 years for all musculoskeletal conditions. Of those, 32.4% were for spine disorders, with 21.2% for low back pain and 11.1% for neck pain, mostly chronic (80.6% and 72.7%, respectively). Women were more likely than men to be referred for both low back pain (odds ratio = 1.36, 95% confidence interval = 1.34–1.38, *p* < 0.001) and neck pain (1.40, 1.37–1.43, *p* < 0.001). All referral rates increased with age. The most common treatment provided by physical therapists for neck and low back pain was education and advice for an active lifestyle.

**Conclusions:**

This study provides comprehensive data that highlight significant trends related to age, acuteness, and sex. Chronic low back and neck pain are the predominant reasons for physical therapy referrals, particularly among women and older adults. Physician referrals for neck and low back pain aligned with the epidemiology of such conditions in the Israeli population, underscoring the need for targeted rehabilitation strategies, early intervention programs, and effective healthcare service planning.

## Background

Musculoskeletal conditions, especially neck and back pain, contribute greatly to the global disease burden [1, 2]. Neck and back pain are the fourth greatest contributors to disability-adjusted life years, and older individuals with such pain tend to have shorter lifespans than those without such conditions. Musculoskeletal conditions affect more than half of adults, and this prevalence increases to three-quarters among individuals aged ≥ 65 years. Notably, the prevalence of these conditions surpasses that of hypertension, diabetes, and cardiovascular disease [2].

A systematic review by Hoy et al. [3] revealed a wide range of neck pain prevalence rates (PRs) per year (1-year prevalence) in different studies, spanning from 4.8 to 79.5%, with a mean of 25.8%. The corresponding rates for low back pain (LBP) ranged from 0.8 to 82.5%, with a mean of 38.1% [3–5]. This heterogeneity poses challenges regarding the comparison and evaluation of results across different studies. It can be attributed to differences in methodologies, variations in the definitions of chronic back or neck pain, and disparities in data collection and calculation methods [3–5].

Although the anatomical regions requiring physical therapy may vary according to the population and regional healthcare trends, musculoskeletal problems consistently rank among the most frequent reasons for referral to physical therapy, particularly for conditions affecting the neck and back [6]. Understanding the characteristics of patients referred to physical therapy is crucial to optimize healthcare planning and resource allocation, especially for prevalent conditions such as neck pain and LBP. Despite the widespread need for these services, limited empirical data exist on referral patterns and the factors that influence them [3, 4, 7, 8].

In many healthcare systems, including Israel’s, physical therapy services are typically accessed through physician referrals, as reimbursement policies frequently require a physician’s recommendation [9]. Additionally, a significant gap in knowledge exists regarding the proportion of referrals related to back pain in comparison to the entirety of physical therapy referrals and how this correlates with diverse clinical and sociodemographic factors.

In this study, we aimed to investigate physician referral patterns for physical therapy across different age and sex groups, with a focus on neck pain and LBP. Additionally, we explored the therapeutic interventions provided by physical therapists for these conditions. Thereby, we sought to gain a deeper understanding of the characteristics of referred patients and the types of interventions they received. We intended to highlight trends and patterns that can inform healthcare planning, support the development of targeted intervention strategies, and optimize resource allocation for physical therapy services.

Considering the substantial impact of neck and back pain on quality of life and the associated costs [10], this study provides valuable insights into referral patterns and patient management for these common musculoskeletal conditions.

## Methods

### Aim

To investigate physician referral patterns for physical therapy across different age and sex groups, with a focus on neck pain and LBP; to explore the therapeutic interventions provided by physical therapists for these conditions; The findings will help identify trends and patterns that can inform healthcare planning, guide intervention strategies, and optimize resource allocation in physical therapy services.

### Study design

This was a retrospective, population-based, cross-sectional study of data in a Health Maintenance Organization (HMO) database. The Institutional Review Boards of Clalit Health Care Services, Israel (#0016-15-COM) and the Faculty of Social Welfare & Health Studies, University of Haifa, Israel (056/15) approved the study protocol.

### Data source

The study was conducted using the Ambulatory Medical Care Survey of the Clalit Health Services database, which is a public HMO and the largest health service organization in Israel. This organization provides health care services for 52.5% of the Israeli population (over 4,100,000 people).

Data collection from Clalit Health Services was conducted in 2015 and included referral information spanning 6 years. Referrals for physical therapy were defined as scheduled treatment appointments at physical therapy clinics. In this study, the term “referrals” only includes patients who attended and received physical therapy treatment at one of the Clalit Health Services outpatient clinics. The inclusion criteria encompassed all insured patients in the Clalit Health Services database (aged *≥* 20 years) who sought physical therapy for neck pain and LBP, and no exclusion criteria were used. Data were obtained from electronic patient records of 100 physical therapy outpatient clinics, all of which were public clinics operated by Clalit Health Services. No private clinics or institutions were included. These records contain demographic information (e.g., age and sex) and medical history (e.g., examinations, treatments, and medical conditions), which can be automatically extracted from the database.

We analyzed referrals for physical therapy related to musculoskeletal disorders over 6 years, focusing on spinal issues, including neck pain and LBP. The 6-year timeframe was selected to ensure that the study captured a sufficiently long period to observe trends and fluctuations in referral patterns while balancing feasibility and data availability. This duration provides a representative sample of recent practice patterns and enables us to assess the stability of referral rates over time. We aimed to minimize potential biases that could arise from using shorter timeframes, such as the influence of temporary changes in clinical guidelines or health policies.

The following parameters were recorded and further analyzed: age, sex, specific diagnosis (musculoskeletal pain and area of the body), and the acute or chronic nature of the pain. Acute pain was defined as pain that lasted less than 6 weeks, subacute pain as pain that lasted between 6 weeks and 3 months, and chronic pain as pain that persisted for longer than 3 months [11]. Additionally, we analyzed data related to the type of treatment provided by the physical therapist for the complaint.

### Statistical analysis

We employed descriptive statistics to summarize the patients’ age, sex, stage of condition, and specific diagnoses. To gain insight into the patterns of physical therapy referrals across different age groups, we employed a combination of descriptive statistics and logistic regression analyses. PRs (%) of referrals for neck pain and LBP were calculated according to age group and sex. The computation of these rates involved dividing the number of referrals (separately for neck pain and LBP) for each sex within a specific age group by the total count of insured individuals in that age group and sex. Importantly, these PRs were calculated for the entire insured population aged ≥ 20 years, enabling a comprehensive examination of trends and patterns. Z-tests were conducted to assess the significance of differences in PRs between women and men within the same age group. When the absolute value of Z exceeds 1.96, the associated p-value is below 0.05.

Logistic regression analyses were used to assess the relative likelihood of patients of a specific age group being referred to physical therapy compared to the total sample. Additionally, we analyzed the PRs of different treatment protocols (%) used by physical therapists according to referrals for neck pain and LBP.

## Results

The total number of referrals in the database for physical therapy over 6 years was 1,593,592 for all musculoskeletal cases, with an annual average of 265,600 referrals. The PR of referral for all musculoskeletal cases was 64.7/1,000 (265,600 referrals per year of the 4,105,445 insurance cases).

The lower back and neck were the most prevalent anatomical locations related to referral for physical therapy. Of the 1,593,592 referrals, 338,526 (21.2%) and 177,413 (11.1%) were associated with LBP and neck pain, respectively. The remaining 67.6% of referrals (1,077,653 of the 1,593,592) were for other musculoskeletal medical diagnoses (knee pain and shoulder pain, among others).

### Acute and chronic symptoms

Among the referrals associated with neck pain and LBP, 65,836/338,526 (19.4%) and 48,423/177,413 (27.3%) pertained to acute diagnoses of LBP and neck pain, respectively.

The most common acute referrals were for whiplash injuries; 25.0% and 17.9% of all referrals for neck pain (44,410 of the 177,413) and LBP (60,560 of the 338,526), respectively, were for such injuries obtained during a vehicular accident, whereas other reasons for referral included surgical interventions and workplace accidents.

Among the referrals associated with neck and back pain, 80.6% and 72.7% of referrals for LBP and neck pain were chronic cases, respectively.

### Referrals for neck pain

The average annual PR of referrals for neck pain was 7.2 per 1,000 persons (177,413 referrals over 6 years of the 4,105,445 insurance cases).

#### Age

**Fig. 1:**
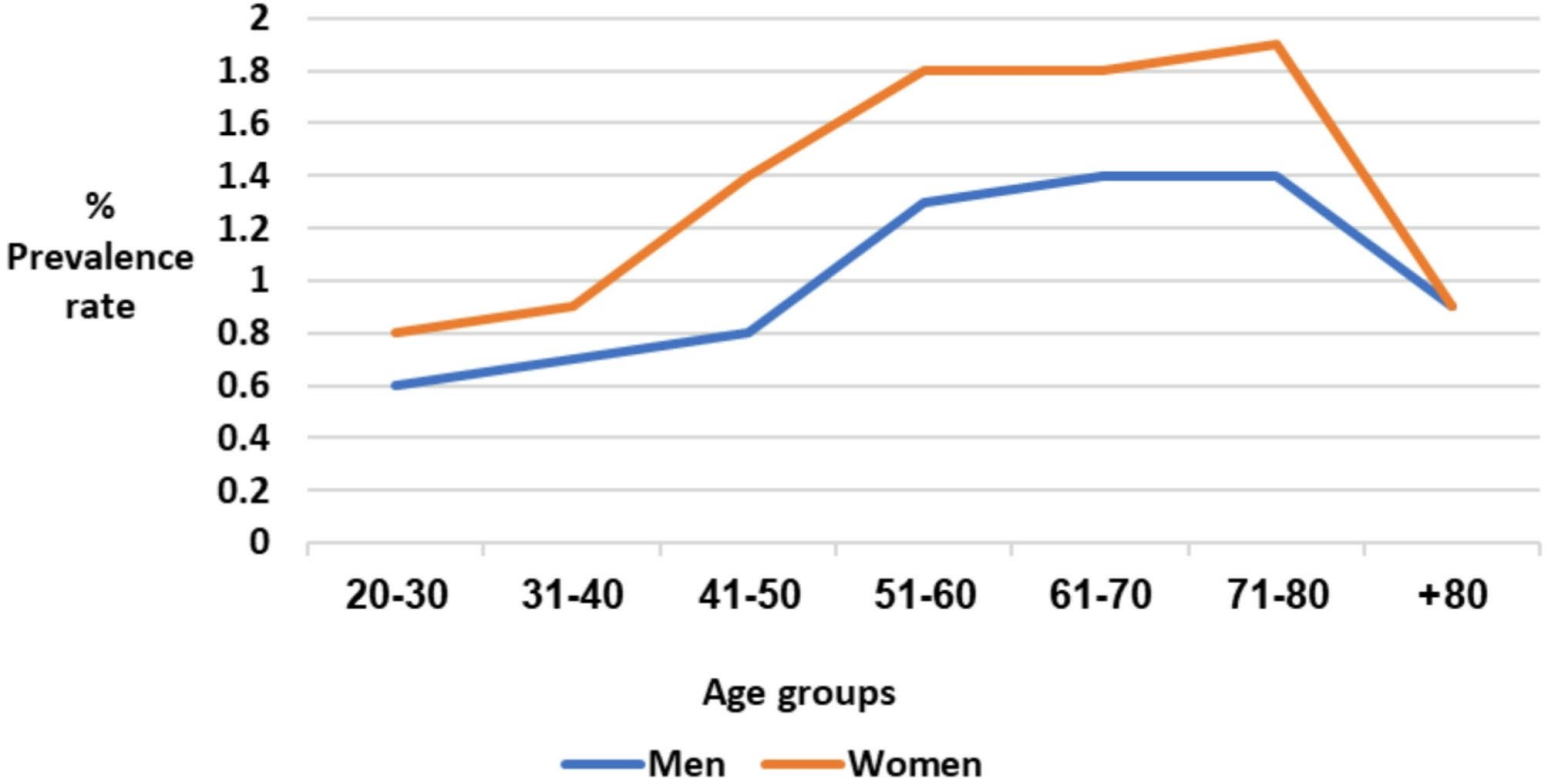
Referrals for neck pain based on sex and age group

A gradual increase in the PR of neck pain referrals from the third to the eighth decades of life was observed. However, in the ninth decade of life, this trend reversed, and the PR of referrals declined. Notably, a considerable increase in the PR of referrals for neck pain was observed from the fifth decade of life for women and the sixth decade for men. Following this increase, the rate stabilized until the age of 80 years (Table 1; Fig. 1).

**Table 1:** Distribution of prevalence rates (%) of referrals for neck pain by age and sex

Age group (years)	Men insured in HMO	Men average annual referrals ^a^(%)	Women insured in HMO	Women average annual referrals ^a^(%)	All insured in HMO	All insured average annual referrals (%)	Difference between men and women Z ratio	Neck pain compared with the total	
								OR (*p*)	95% CI
**20–30**	295,626	1707 (0.6%)	299,513	2357 (0.8%)	595,139	4064 (0.7%)	9.81***	0.61 (*p* < 0.001)	0.59, 0.63
**31–40**	272,624	1919 (0.7%)	267,803	2484 (0.9%)	540,427	4404 (0.8%)	9.14***	0.73 (*p* < 0.001)	0.71, 0.76
**41–50**	208,111	1636 (0.8%)	215,349	2910 (1.4%)	423,460	4546 1%)0.1)	17.84***	0.97 (*p* = 0.045)	0.94, 0.99
**51–60**	164,013	2206 (1.3%)	210,743	3720 (1.8%)	374,756	5927 (1.6%)	7.85***	1.43 (*p* < 0.001)	1.39, 1.48
**61–70**	146,517	2020 (1.4%)	177,782	3130 (1.8%)	324,299	5150 (1.6%)	11.01***	1.44 (*p* < 0.001)	1.40, 1.48
**71–80**	97,332	1385 (1.4%)	125,201	2329 (1.9%)	222,533	3713 (1.7%)	7.98***	1.51 (*p* < 0.001)	1.46, 1.57
**> 80**	52,582	466 (0.9%)	79,150	679 (0.9%)	131,732	1145 (0.9%)	-0.54	0.78 (*p* < 0.001)	0.74, 0.83
**Total** **(> 20 years)**	1,236,805	**11,339** **(0.9%)**	1,375,541	**17,609** **(1.3%)**	2,612,346	**28,949** **(1.1%)**	28.0***		

Significant: ***p < 0.001

^a^Annual number of referrals divided by number of insured persons in the same age group.

HMO, health management organization (Clalit Health Services), CI, confidence interval; OR, odds ratio

#### Sex

Women were more likely than men to be referred for physical therapy for neck pain (odds ratio = 1.40, *p* < 0.001, 95% confidence interval = 1.37–1.43).

Moreover, across all age groups, spanning from the third to the eighth decades of life, the PR of neck pain referrals was consistently higher among women than among men. However, in the age group of ≥ 80 years, the PR of referrals was comparable between the sexes (Table 1; Fig. 1).

Additionally, the likelihood of patients of a specific age group being referred for physical therapy compared to the total sample was analyzed. For neck pain, patients aged 20–40 or 80 + years were below average in terms of referrals, whereas those aged 51–80 years were above average (Table 1).

### Referrals for LBP

The average annual PR of referrals for LBP was 13.7 per 1,000 persons (338,526 referrals over 6 years of the 4,105,445 insurance cases).

#### Age

The PR for LBP referrals gradually increased with advancing age. Patients aged 20–50 years were below average regarding referrals, whereas those aged 51 + years were above average. The most substantial increase occurred among women between the fourth and eighth decades of life, during which the referral rate tripled. Among men, a considerable referral surge began in the fifth decade of life, continuing until the age of 80 years, with the referral rate more than doubling. Beyond the eighth decade of life, a noticeable decrease in the PR of referrals for LBP was observed for both sexes.

#### Sex

**Fig. 2:**
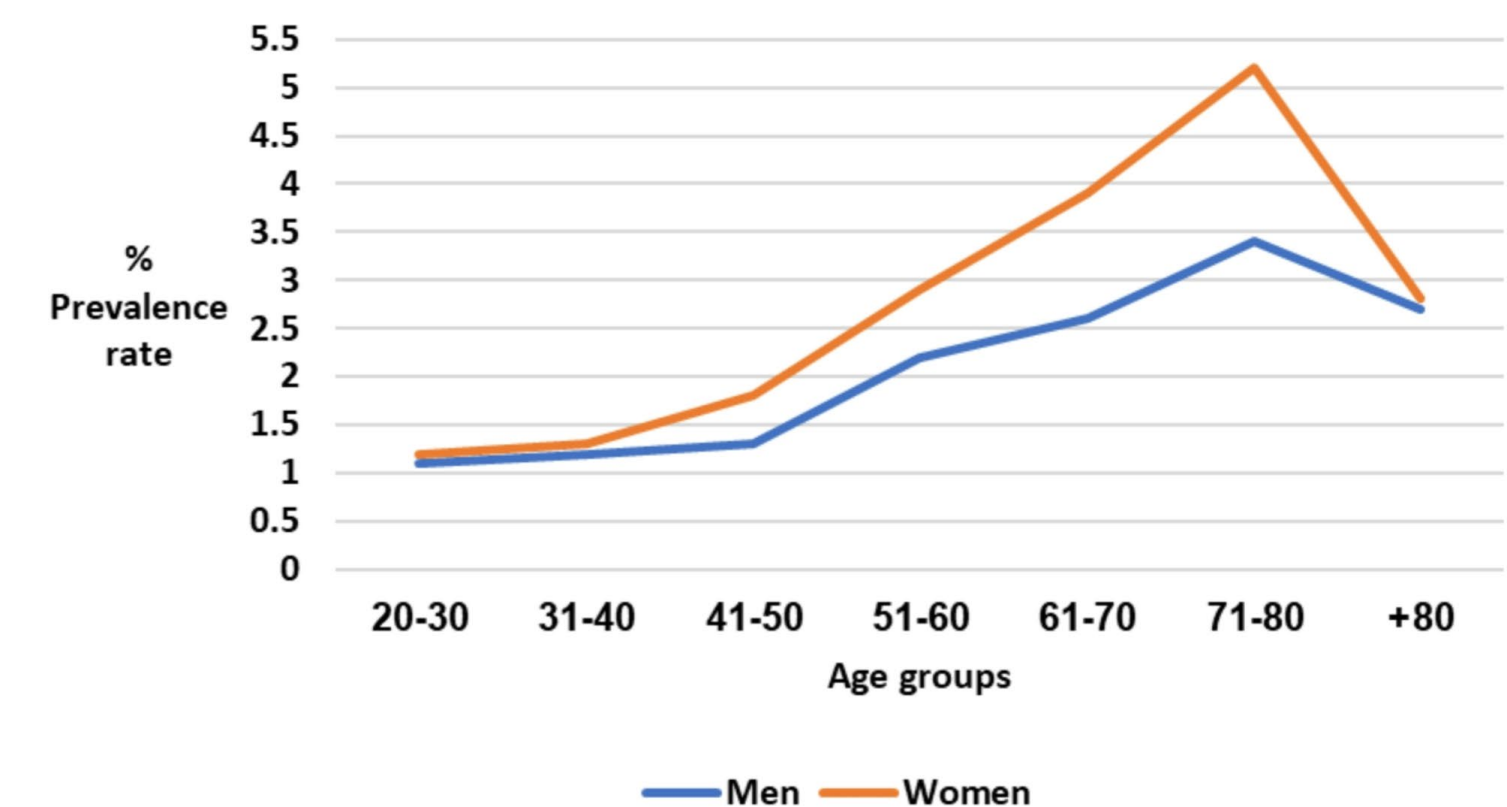
Referrals for low back pain based on sex and age group

Women were more likely than men to be referred for physical therapy for LBP (odds ratio = 1.36, *p* < 0.001, 95% confidence interval = 1.34–1.38). The PR for LBP was consistently higher among women than among men, from the fifth to the eighth decades of life. In the younger (20–40 years) and older (> 80 years) age groups, no statistically significant differences were identified in the PR of referrals for LBP between the sexes (Table 2; Fig. 2).

**Table 2:** Distribution of prevalence rates (%) of referrals for low back pain by age and sex

Age group (years)	Men insured in HMO	Men average annual referrals ^a^(%)	Women insured in HMO	Women average annual referrals ^a^(%)	All insured in HMO	All insured average annual referrals (%)	Difference between men and women Z ratio	Low back pain compared with the total	
								95% CI	95% CI
**20–30**	295,626	3377 (1.1%)	299,513	3466 (1.2%)	595,139	6844 (1.1%)	0.53	0.55 (*p* < 0.001)	0.53, 0.56
**31–40**	272,624	3383 (1.2%)	267,803	3390 (1.3%)	540,427	6773 (1.3%)	0.82	0.60 (*p* < 0.001)	0.58, 0.61
**41–50**	208,111	2697 (1.3%)	215,349	3850 (1.8%)	423,460	6547 (1.5%)	12.9***	0.74 (*p* < 0.001)	0.72, 0.76
**51–60**	164,013	3628 (2.2%)	210,743	6198 (2.9%)	374,756	9826 (2.6%)	10.7***	1.27 (*p* < 0.001)	1.24, 1.30
**61–70**	146,517	3867 (2.6%)	177,782	6948 (3.9%)	334,299	10,815 (3.2%)	23.4***	1.58 (*p* < 0.001)	1.54, 1.61
**71–80**	97,332	3342 (3.4%)	125,201	6487 (5.2%)	222,533	9829 (4.4%)	19.9***	2.18 (*p* < 0.001)	2.13, 2.23
**> 80**	52,582	1419 (2.7%)	79,150	2240 (2.8%)	131,732	3659 (2.8%)	1.42	1.35 (*p* < 0.001)	1.30, 1.39
**Total** **(> 20 years)**	1,236,805	**21,713** **(1.8%)**	1,375,541	**32,579** **(2.4%)**	2,612,346	**54,292** **(2.1%)**	34.6***		

Significant: ***p < 0.001

^a^Annual number of referrals divided by the number of insured persons in the same age group.

HMO, health management organization (Clalit Health Services), CI, confidence interval; OR, odds ratio

### Treatment protocol for neck and LBP referrals

The most common treatment provided by physical therapists for neck pain and LBP was education and advice for an active and healthy lifestyle (19.7% and 21.0%, respectively). The second most common treatment for neck pain was manual therapy (17.7%), followed by specific therapeutic exercises (16.8%) and thermotherapy and electrotherapy (13.6% each). The second most common treatment for LBP was specific therapeutic exercises (20.8%), followed by electrotherapy (16.6%) and manual therapy (13.0%) (Table 3).

**Table 3:** Treatment session distribution for neck and low back pain

Type of treatment	Low back pain	Neck pain
	Treatment sessions, *n* (%)	
**Education and advice**	216,212 (21.0%)	118,590 (19.8%)
**Manual therapy**	134,139 (13.0%)	106,390 (17.7%)
**Therapeutic exercise**	213,454 (20.8%)	100,849 (16.8%)
**Thermotherapy**	110,235 (10.7%)	81,359 (13.6%)
**Electrotherapy**	170,406 (16.6%)	81,519 (13.6%)
**Ultrasound**	39,315 (3.8%)	28,153 (4.7%)
**Traction**	19,249 (1.9%)	12,107 (2.0%)
**Tapping**	6,423 (0.6%)	3,302 (0.6%)
**Others**	119,093 (11.6%)	67,819 (11.3%)
**Total**	**1**,**028**,**526**	**600**,**088**

## Discussion

In this study, we investigated physician referrals of patients to physical therapy in Israel’s largest health service organization. The main findings were that 32.4% of physician referrals of patients for physical therapy were linked to spine disorders, comprising 21.2% and 11.1% for LBP and neck pain, respectively. Rates of neck pain referrals in a previous study were higher (13.6% and 20.0% in the United States and the Netherlands, respectively) [12]. Regarding LBP referrals, in Denmark and the United States, patients with LBP comprise approximately 30% of all those who visit physical therapists [12, 13]. The reported rate in the Netherlands is 21.9%, which closely aligns with our results [12]. Differences in referral rates among countries may be attributed to factors, including cultural distinctions, types of medical insurance, and waiting times for physical therapy treatment. These variations underscore the difficulty of comparing the reasons for referral across studies [14]. Further studies are required to elucidate the nature of these variations.

Our investigation identified higher PRs of referrals for neck pain and LBP among women than among men across most age groups. Particularly, the 51–80-year age groups exhibited above-average referrals for both neck pain and LBP among women. The rate ratio between women and men for neck pain remained around 1.5, whereas that for LBP was approximately 1.3. These sex-based observations align with those of earlier studies and underscore the necessity for tailored healthcare policies that address sex differences [15–18].

Notably, numerous studies have consistently revealed a higher incidence of both neck pain and LBP among women than those among men [19–24]. This disparity can be attributed to a combination of factors. Occupational influences, such as the types of jobs women typically engage in, frequently involve repetitive tasks or sustained postures, which may increase susceptibility to musculoskeletal disorders in both the neck and lower back regions [25]. Biological factors, including differences in body mass, muscular capacity, and hormonal fluctuations, may also contribute to the heightened prevalence of these conditions [26, 27]. Additionally, women are generally more likely to seek healthcare services for pain-related conditions [28, 29], leading to higher referral rates for physical therapy. Psychosocial factors, including stress and caregiving roles, may further exacerbate chronic pain in both the neck and lower back [30, 31]. Understanding and addressing these multifaceted contributors is crucial for effective management and targeted interventions aimed at reducing sex-based differences in physical therapy utilization and outcomes.

The substantial increase in the referral rate for neck pain observed in this study from the fifth decade of life among women and the sixth decade among men corresponds with the results of previous studies that revealed a sharp increase in neck and upper-limb pain rates from the fourth decade of life among women and the fifth decade of life among men [32, 33].

In our analysis of the rate of referrals for LBP regarding age and sex, we observed a gradual increase with age. This increase became more pronounced from the fourth decade among women and the fifth decade among men, continuing until the eighth decade of life, with a doubling to tripling of the rate. From the eighth decade onward, the referral rate notably declined for LBP in both sexes. This decline may be attributed to decreased mobility, a lower adoption of health-promoting behaviors [34], and increased comorbidities among older adults, which can limit their ability or willingness to seek physical therapy services. Despite the decrease in this age group, the rate remained higher than that among patients in their 20s to 40s.

Our study revealed that 80.6% and 72.7% of referrals for LBP and neck pain, respectively, were associated with chronic pain. The cases of acute neck pain primarily resulted from whiplash injuries. Direct comparisons with previous studies are challenging owing to variations in study focus and methodology. Breivik et al. [35] reported a 17% incidence of chronic pain in the Israeli population, which was lower than the European average of 19%. In Israel, approximately one-third (33%) of patients with chronic pain received physical therapy, differing from those in Sweden (55%), the Netherlands (52%), Norway (47%), and the European average (21%) [35]. Notably, in Israel, the waiting time for physical therapy for chronic conditions in HMOs ranges from 3 to 6 months. Thus, some of the patients who are referred for physical therapy may not follow through.

Our investigation disclosed an average of 66.4 referrals for outpatient physical therapy for musculoskeletal pain per 1,000 persons. Physicians consistently referred patients for physical therapy for neck and LBP, aligning with observations in previous studies [6, 18, 36, 37], although the referral rate for musculoskeletal pain in one study was lower, at 42.9 referrals per 1000 people [36]. Notably, cultural differences, variations in medical insurance, and differences in waiting times for physical therapy might have contributed to the disparities in referral rates across countries, necessitating further in-depth research.

Our study revealed that the predominant treatment modality prescribed by physical therapists for chronic neck pain and LBP was education and advice for an active and healthy lifestyle. The second most common treatment for neck pain was manual therapy, followed by specific exercises, electrotherapy, and thermotherapy. For LBP, the second most prevalent treatment was specific exercises, followed by electrotherapy and manual therapy. However, divergences were observed in other countries. For example, in the Netherlands, an active therapeutic approach is followed, emphasizing specific exercises and manual therapy and downplaying electrotherapy [12]. Some similarities with studies in other countries were also observed. For LBP, more than 95% of physical therapists in Denmark recommended activity and work that was partly or strictly in line with clinical practice guidelines (CPGs) [37]. For neck pain, 99.1% of patients in America received advice for home exercise and to keep an active lifestyle; 52.3% received manual therapy; 12.5% received electrotherapy; and 89.3% partook in scapular resistance exercises [38].

The disparities in therapeutic interventions between studies and populations may stem from differences in culture, economic resources, treatment waiting times, personnel expertise, educational training, and the dichotomy between public and private healthcare systems [39]. Importantly, the abovementioned treatments align with established CPGs for neck pain and LBP [40, 41]. National guidelines for chronic LBP recommend progressive aerobic exercises with a moderate to high level of evidence [42, 43]. Similarly, thoracic and cervical manipulation or mobilization and mixed exercises, encompassing stretching, strengthening, endurance training, and aerobic conditioning, have been advocated for neck pain [41, 44, 45]. These aligned practices underscore a commitment to evidence-based care and suggest a foundation for clinical consensus in managing neck pain and LBP within the HMO. A recent systematic review of the impact of implementing and adhering to CPGs revealed significant benefits regarding reduced healthcare utilization. It has resulted in decreases in costs, the total number of healthcare visits, medication use, and procedural interventions [46].

### Limitations

Analyzing retrospective data from HMOs has certain limitations. HMOs may serve distinct demographic groups with unique health characteristics, potentially introducing a selection bias [47]. To address this, we sourced data from an HMO covering 52.5% of the Israeli population, which improves the generalizability of our findings. Additionally, the reliability of retrospective studies depends on the completeness of electronic health records; however, the data used in this study were all electronically recorded, minimizing concerns about missing information. Furthermore, HMOs may have specific healthcare utilization patterns that influence the observed associations and limit external validity [48]. Our data may differ from those in countries where physical therapy is sought privately [40]. Patients opting for private physical therapy owing to long public waiting times are an unmeasured variable that might have influenced the results. We did not have access to private clinic data during the study period.

Another limitation is that the dataset includes only initial referrals and does not capture the number of treatment sessions or distinguish between initial and repeat referrals, which prevents the assessment of treatment adherence and recurrent referral trends. Future studies should address these issues to better understand referral patterns.

Additionally, the data available from Clalit Health Services allowed access only to predefined treatment categories such as electrical therapy, manual therapy, exercise-based therapy, lifestyle guidance, neurological therapy, and vestibular therapy. While this predefined data provides a general understanding of treatment categories, the lack of access to free-text descriptions limits our ability to evaluate nuanced variations in treatment protocols and their specific effectiveness. We acknowledge that this limitation impacts the ability to comprehensively assess treatment effectiveness.

Finally, this study was conducted before the coronavirus disease 2019 pandemic, which might have affected the prevalence and management of musculoskeletal conditions. Despite these limitations, the data provide valuable insights into real-world healthcare practices and outcomes.

### Future studies

Future research should explore the influence of cultural factors on physician referral patterns for musculoskeletal conditions, the impact of health insurance disparities on referral rates, and the effectiveness of early physical therapy interventions for neck pain and LBP. Additionally, collecting data on the number of treatment sessions completed by each patient is crucial to gain a deeper understanding of treatment adherence and utilization. This will help identify potential differences in treatment outcomes between the sexes and improve the planning of targeted interventions.

Further research should explore treatment protocols in greater detail. While this study focused on CPGs, it did not examine specific treatment protocols for LBP and neck pain. Physiotherapy for such conditions typically adheres to general recommendations, such as education for healthy lifestyle and exercise-based interventions, with considerable reliance on the clinical judgment of individual therapists. Variability in treatment approaches across therapists and clinics, highlights the need to investigate the impact of CPG adherence and the potential benefits of standardizing protocols on treatment outcomes for musculoskeletal conditions. Addressing these areas can enhance the tailoring of interventions and advance global musculoskeletal healthcare practices.

### Policy implications and recommendations

The study was conducted using the Ambulatory Medical Care Survey of Israel’s largest health service organization. The results of the study are important for policymakers and health care providers to create effective prevention and treatment programs within Isreali healthcare systems.

The study has various important *policy implications*. (1) High prevalence of referrals for LBP and neck pain: The high prevalence of referrals for physical therapy for musculoskeletal disorders, particularly LBP and neck pain, indicates a substantial burden on healthcare resources. This suggests a need for targeted interventions to manage and reduce the incidence of these conditions. (2) Chronic nature of referrals: A large proportion of referrals for LBP and neck pain are for chronic conditions. This highlights the need for long-term management strategies and possibly for preventative measures to reduce the progression from acute to chronic pain. (3) Age-related trends: The study indicates that referrals for LBP and neck pain increase with age, particularly among individuals in their fifth to eighth decades of life. This demographic trend suggests that healthcare policies should focus on older populations for early intervention and management of musculoskeletal conditions. (4) Sex differences: Women are more likely than men to be referred for physical therapy for both neck pain and LBP. This disparity suggests that sex-specific strategies may be necessary to address the unique needs of women in managing musculoskeletal pain.

Our study leads us to make several *recommendations*. (1) Preventive programs: Implement preventive programs aimed at reducing the incidence of musculoskeletal disorders, particularly targeting younger populations to prevent the development of chronic conditions. This could include workplace ergonomics training, physical activity promotion, and educational campaigns on proper body mechanics. (2) Chronic pain management: Develop and promote comprehensive chronic pain management programs. These should include multidisciplinary approaches involving physical therapy, pain management specialists, and mental health support to address the multifaceted nature of chronic pain. (3) Targeted interventions for older adults: Given the increase in referrals with age, particularly for individuals in their fifth to eighth decades, targeted interventions should be prioritized. Older adults often face mobility limitations, higher reliance on public healthcare, and delayed access to treatment, which can lead to prolonged disability. To address this, policies should include age-specific physical therapy programs, fall prevention strategies, and regular screening for early detection of musculoskeletal issues, ensuring timely and effective care [49].

4) Sex-specific strategies: Women are referred for physical therapy at higher rates than men, suggesting the need for tailored interventions that address biomechanical, hormonal, and psychosocial factors influencing musculoskeletal pain. Additionally, socioeconomic and geographic barriers can further limit access for certain populations, particularly low-income and rural women. Expanding community-based services, integrating telerehabilitation, and improving access to specialized programs can help close these gaps and ensure equitable care. 5) Data collection and monitoring: Enhance data collection efforts to include socioeconomic status and other relevant demographic factors. This will enable a more comprehensive analysis of the determinants of musculoskeletal referrals and help in designing more effective public health interventions. 6) Education and training: Increase education and training for healthcare providers on the latest evidence-based practices for the management of musculoskeletal disorders. This includes training on the recognition of early signs of chronic pain and implementation of appropriate referral pathways.

The above recommendations should be implemented in the different public healthcare systems in collaboration with the Ministry of Health, making these measures available to the entire population. Effective policies will integrate the expert knowledge in an ever-changing technological, epidemiological, social, and economic context to ensure long-term success [50].

### Healthcare planning

The study results highlight the high prevalence of chronic LBP and neck pain in the population referring to physical therapy treatment, raising gaps in the healthcare system that need to be addressed. The healthcare system must improve physical therapy availability by addressing the shortage of physical therapists in public healthcare. This shortage leads to long waiting lists, therapists overload and burnout, and decreased care quality [51]. Israel has fewer physiotherapists (~ 7–8 per 10,000) and longer wait times (months) due to referral requirements, compared to the Netherlands (~ 19–20 per 10,000) with direct access and shorter wait times (days to weeks) [52, 53]. Expanding workforce capacity, extending clinic hours, increasing the number of physical therapists, and increasing job opportunities with competitive salaries can help address these challenges [54, 55].

Shorter wait times and greater access to physical therapy can prevent conditions like LBP from becoming chronic, reducing hospitalizations and disability-related expenses [56, 57]. Early intervention is also linked to higher patient satisfaction, lower healthcare costs, and a reduced need for unnecessary imaging, medications, and surgeries [58–60]. Integrating various intervention modalities such as personal physical therapy, group therapy, telerehabilitation, and preventive exercise programs, can enhance recovery rates while minimizing the burden on the healthcare system [61–63].

Another way to increase healthcare availability is through telerehabilitation, which should be further integrated into healthcare services. Telerehabilitation has emerged as a key advancement in physical therapy, particularly for individuals in remote areas or those with transportation challenges. By utilizing digital platforms, telerehabilitation facilitates remote consultations and therapy, ensuring continuous patient care despite geographical barriers. Studies confirm its effectiveness in expanding treatment availability and improving outcomes [64–66].

Direct access to physical therapy, allowing patients to seek treatment without a physician’s referral, has proven successful in healthcare systems such as the Netherlands and the U.S., where it reduces delays and improves patient outcomes. In Israel, referral policies vary: Maccabi health care allows direct access for orthopedic cases, while Clalit requires a physician’s referral. The U.K. still mandates general practitioner referrals for National health services (NHS( physiotherapy, though self-referral is expanding. In the U.S., all states allow some form of direct access, though insurance often requires physician approval [55, 59, 60]. Studies indicate that direct access lowers healthcare costs by minimizing unnecessary physician consultations, aids in preventing chronic conditions, improves patient outcomes, decreases the number of needed treatments, and further decreases healthcare costs while improving care efficiency [67].

By promoting an integrated primary care system with direct access, telerehabilitation, and early intervention strategies, healthcare providers can ensure timely treatment, reduce unnecessary delays, and optimize economic and clinical outcomes.

### Economic justification and funding

Implementing preventive programs, telerehabilitation, and direct access to physical therapy is crucial for improving patient outcomes and significantly reducing long-term healthcare costs. Early physiotherapy intervention for musculoskeletal conditions lowers the risk of chronic pain, reducing reliance on costly imaging, medications, and surgeries, while also preventing hospitalizations and disability-related expenses [56, 58, 68, 69].

Chronic pain, including back and neck pain, imposes a substantial economic burden, with estimated costs of €12 billion annually in Europe, 80% of which is due to productivity loss [10]. Prolonged wait times for physiotherapy exacerbate these costs by increasing healthcare expenditures and workplace absenteeism, contributing to lost productivity [56]. Expanding workforce capacity, integrating telerehabilitation, and allowing direct access can mitigate these losses by improving treatment efficiency and workforce participation [55].

Prioritizing early physiotherapy access and digital health solutions will create a more cost-efficient, patient-centered healthcare system, optimizing both economic and clinical outcomes. These approaches can help mitigate health disparities by improving access to care, reducing the burden of chronic conditions, and enhancing overall population health. The economic burden of health disparities related to socioeconomic status in Israel is significant, with estimates suggesting a cost of 0.7–1.6% of Israel’s gross domestic product due to productivity losses from health impairments [70]. Funding these initiatives should involve a collaborative effort between HMOs, the Ministry of Health, and private insurers, as reducing absenteeism, disability, and long-term care needs benefits both the healthcare system and the broader economy [71].

## Conclusions

This study provides comprehensive data on physician referral patterns for physical therapy related to neck pain and LBP, highlighting significant trends based on age, acuteness, and sex. The findings confirm that chronic LBP and neck pain are the leading reasons for referrals, particularly among women and older adults. This underscores the need for targeted rehabilitation strategies, early intervention programs, and effective healthcare service planning.

To address these challenges, healthcare policies should prioritize: (1) Preventative and early intervention strategies to reduce chronic pain progression; (2) Improved accessibility to physical therapy, including direct access, integrated telerehabilitation, and community-based models; (3) Age-specific and sex-responsive interventions to address demographic disparities in referral patterns.

Implementing these strategies in healthcare planning could improve the management of musculoskeletal pain, leading to better patient outcomes and reduced healthcare costs. A collaborative effort involving policymakers, healthcare providers, and insurers is crucial to ensuring that these recommendations are effectively integrated into Israel’s healthcare framework.

## Data Availability

The datasets used and/or analyzed during the current study are available from the corresponding author upon reasonable request.

## Abbreviations

CPG: Clinical practice guideline
HMO: Health maintenance organization
LBP: Low back pain
PR: Prevalence rate
